# Dysfunction of NMDA receptors in Alzheimer’s disease

**DOI:** 10.1007/s10072-016-2546-5

**Published:** 2016-03-12

**Authors:** Yan Zhang, Peiyao Li, Jianbo Feng, Minghua Wu

**Affiliations:** Hunan Provincial Tumor Hospital and the Affiliated Tumor Hospital of Xiangya Medical School, Central South University, Changsha, 410013 Hunan China; Cancer Research Institute, School of Basic Medical Science, Central South University, Changsha, 410078 Hunan China; Key Laboratory of Carcinogenesis and Cancer Invasion, Ministry of Education, Changsha, 410078 Hunan China; Key Laboratory of Carcinogenesis, Ministry of Health, Changsha, 410078 Hunan China

**Keywords:** NMDA receptors, Extrasynaptic, Tripartite synapse, Synaptic transmission, AD

## Abstract

*N*-methyl-d-aspartate receptors (NMDARs) play a pivotal role in the synaptic transmission and synaptic plasticity thought to underlie learning and memory. NMDARs activation has been recently implicated in Alzheimer’s disease (AD) related to synaptic dysfunction. Synaptic NMDARs are neuroprotective, whereas overactivation of NMDARs located outside of the synapse cause loss of mitochondrial membrane potential and cell death. NMDARs dysfunction in the glutamatergic tripartite synapse, comprising presynaptic and postsynaptic neurons and glial cells, is directly involved in AD. This review discusses that both beta-amyloid (Aβ) and tau perturb synaptic functioning of the tripartite synapse, including alterations in glutamate release, astrocytic uptake, and receptor signaling. Particular emphasis is given to the role of NMDARs as a possible convergence point for Aβ and tau toxicity and possible reversible stages of the AD through preventive and/or disease-modifying therapeutic strategies.

## Introduction

Alzheimer’s disease is the most common form of dementia and the most prevalent neurodegenerative disease characterized by cognitive disorder and memory dysfunction in the elderly population, affecting almost 40 million people worldwide [1]. AD progression has been associated with a gradual damage in function and structure in the hippocampus and neocortex, the vulnerable brain areas involved in memory and cognition. AD is characterized by synaptic loss, deposition of Aβ plaques, neurofibrillary tangles (NFTs), and hyperphosphorylated tau. These changes are associated with NMDARs activation and oxidative stress which ultimately results in AD pathology. Besides, Aβ is also reported to trigger NMDA-mediated Ca^2+^ influx, excitotoxicity, and stress-related signaling pathways in neurons which may exacerbate aging-related increases in oxidative stress, impaired energy metabolism, and defective Ca^2+^ homeostasis [2]. The NMDARs are cationic channels gated by the neurotransmitter glutamate which play an essential role in excitatory transmission, synaptic integration, learning and memory in the central nervous system (CNS). NMDAR activation, excessive Ca^2+^ fluxes, and free radical generation are associated with synaptic dysfunction and tau phosphorylation. Excessive amounts of glutamate are associated with intense transient influx of Ca^2+^, leading to mitochondrial functional impairments characterized by activation of the permeability transition pores in the inner mitochondrial membrane, cytochrome c release and depletion of ATP, and simultaneous formation of ROS [3]. Therefore, it seems that proper NMDAR and synapse function are necessary for learning and memory, and improper of NMDAR and synapse function may participate in the pathogenesis of AD at the synaptic level.

## The NMDAR subtypes associate with neurological disorders

**Fig. 1:**
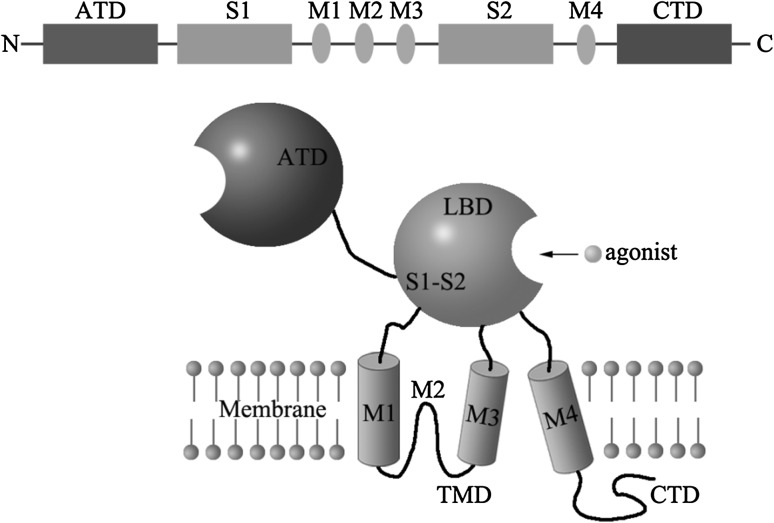
Structure and domain organization of NMDA receptors. Linear representation of the subunit polypeptide chain and schematic illustration of the subunit topology. NMDARs subunits have a modular structure composed of two extracellular domains [the ATD (*green*) and the LBD (*yellow*)]; a TMD (*cyan*) that forms part of the ion channel pore; and an intracellular CTD. The LBD is defined by two segments of amino acids termed S1 and S2. The TMD contains three membrane-spanning helices (M1, M3, and M4) and a membrane re-entrant loop (M2)

All glutamate receptor subunits share a common membrane topology that contain four discrete semiautonomous domains: the extracellular amino-terminal domain (ATD), the extracellular ligand-binding domain (LBD), where glutamate binds to and which created by two regions (S1 and S2) between M3 and M4, the transmembrane domain (TMD) which contains three transmembrane segments (M1, M3 and M4) and a re-entrant pore loop (M2), and an intracellular carboxyl-terminal domain (CTD) which interacts with multiple cytosolic proteins (Fig. 1). The re-entrant M2 loop is part of the channel pore and it contains a critical asparagine residue that determines calcium permeability of the channel and mediates the magnesium blockade [4]. Seven NMDAR subunits have been identified: one NR1, four NR2 (A, B, C and D), and two NR3 (A, B) subunits. NR1 occurs as eight distinct splice variants, likewise, each of the NR2 and NR3 subunits (except NR2A) has several isoforms. Most native NMDARs are heterotetramers containing two glycine-binding NR1 and two glutamate-binding NR2 subunits. The NR1–NR2 dimer is considered to be the basic functional structure in each receptor. The structure of NDMAR forms a Ca^2+^-permeable ion channel. NR3A can also co-assembles with NR1/NR2 to form a receptor complex. Besides, a portion of native NMDARs are triheteromers assembled by two NR1 and two different NR2 subunits [5]. The most NMDARs contain the obligatory subunit GluN1 plus either GluN2B or GluN2A or a mixture of the two.

Expression of NMDAR subunits differentially distribute throughout the brain and change strikingly during development. NR1, NR2A and NR2C are mildly expressed in the temporal region of the cerebral cortex and the hippocampus in the embryonic brain, while in neonatal brain they express widely throughout the brain. The mRNA of the NR1 subunit begins to be expressed in the rat brain at embryonic day 14 and increases gradually until third postnatal week, since then it decreases to adult levels. NR2B subunit is mildly expressed in the hippocampus and the temporal region of the cerebral cortex in fetal brain, becomes hardly expressed in adults [6]. During postnatal stage, NR3A protein level is high, decreases from postnatal week 7 to 21 while NR3B protein expression starts to increase and NR3A remains low while NR3B remains high in adult. In humans, NR1 expression is low during gestation, increases after birth until adolescence and remains high throughout life. NR3A levels are low in embryonic day, increases rapidly after birth and declines gradually into adulthood [7]. The level of NR2B mRNA is significantly lower in the neonate than other age groups. While NR2A mRNA levels remains constant after birth, leading to an age-related increase in NR2A/2B transcript ratio. It is believed that the pre- and postnatal expression changes of NMDAR subunits are important to mediate the synaptic plasticity lifelong. NMDARs are the primary channel that mediates Ca^2+^ signals in hippocampal neurons and contribute to the expression of long-term potentiation (LTP) and long-term depression (LTD) and for synaptic plasticity [8]. Both NR2A and NR2B subunits are critical for the induction of LTP and LTD [9]. So, NMDARs mediate key physiological functions such as learning and memory under normal conditions.

Several SNPs in the NMDAR subunit genes have been shown to implicated in the pathophysiology of neurological disorders, such as AD, schizophrenia, bipolar disorder, and depression. A missense mutation in the coding regions of the GRIN2B was found only in the brains of AD patients, suggesting that close relationship between this mutation of the postsynaptic NR2B-subunit and alteration of synaptic structures. Statistical indicate that multiple coding variants on the risk haplotype containing rs1806201 may play a role in mediating susceptibility to AD [10]. And also the frequency of Ht2-AG haplotype in GRIN3A gene is higher in AD patients indicates that GRIN3A variant may represent a risk in the development of AD [11]. People carrying insCGTT which is a genetic variation of the NMDAR NR3B subunit that inserts four bases within the coding region have a predisposition to schizotypal personality traits [12]. And the GRIN3A R480G variant showed strong association with schizophrenia [13]. GRIN2A mutation of substitution p.N615K is found in a girl with early-onset epileptic encephalopathy. By analysis of the receptor currents of NR1-NR2A with the p.N615K mutation, they found a decrease in Ca^2+^ permeability. So NR2 subunit of NMDARs affected may disturbance electrophysiological balance during development which results in variable neurological phenotypes [14]. In conclusion, NMDARs SNP variants may affect synaptic transmission and plasticity, results in severe neurological disorders associate with defective glutamate transmission.

## The tripartite glutamate synapse

**Fig. 2:**
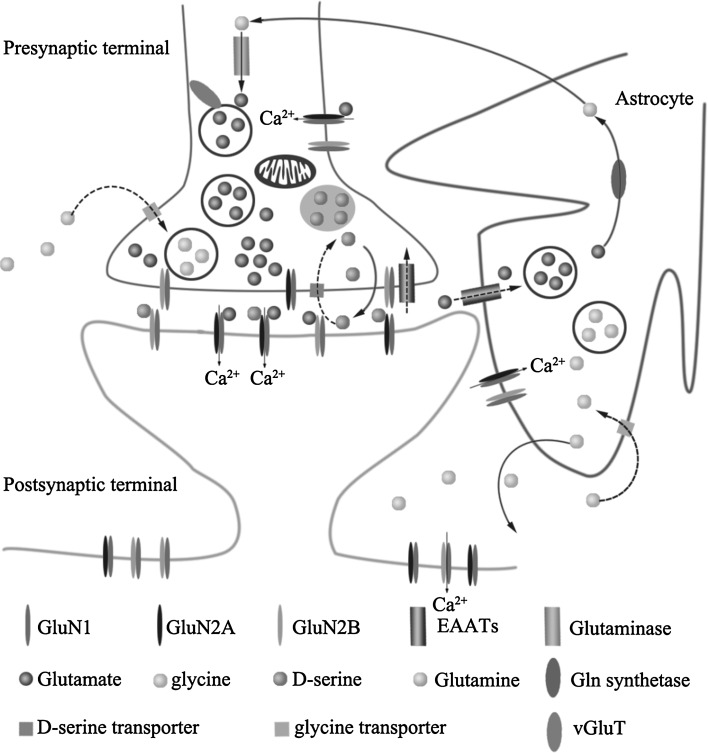
The tripartite glutamate synapse. Neurotransmitter molecules sequester in vesicles of the presynaptic neuron, glutamate is released into the synaptic cleft. Once released, glutamate bind to and activate a variety of ionotropic and metabotropic receptors on postsynaptic and presynaptic neurons as well as glial cells. The activation of NMDARs localized in the postsynaptic membrane leading to Ca^2+^ entry through the NMDARs and the propagation of the action potential. Glutamate can then be taken up by surrounding astrocytes through EAAT1/2 or by the presynaptic terminal through EAAT2/5, and then stored into vesicles. The activation can also be modulated by glycine released from neurons and astrocytes or d-serine released by astrocytes. Both glycine and d-serine can also be taken up by the presynaptic terminal or astrocytes by their respective transporters

The term “tripartite synapse” proposed 20 years ago to describe communication between neurons and astrocytes, includes a presynaptic terminal, a postsynaptic spine, and an astrocytic cell (Fig. 2). Within the tripartite synapse are multiple sites that regulate extracellular glutamate levels and are sensitive to AD-related pathology. Below, the normal physiological processes regulating extracellular glutamate are briefly described, followed by descriptions of how these targets are deregulated in AD. Glutamate is packaged into synaptic vesicles by vesicular glutamate transporters (VGLUTs) presynaptically. Following presynaptic neuronal depolarization, calcium channels open, permitting the influx of calcium and triggering the fusion of vesicles with the membrane, resulting in the exocytosis of glutamate into the synapse [15]. Once released the glutamate can activate a variety of ionotropic and metabotropic receptors on postsynaptic and presynaptic neurons as well as glial cells. In fact, astrocytes may respond to neuronal activity through an elevation of internal Ca^2+^ concentration, which further leads to the release of neurotransmitters able to cause feedback regulation of neuronal activity and synaptic efficacy. Perturbations in the glutamatergic tripartite synapse may underlie the pathogenic mechanisms of AD [16]. Glutamate is cleared from the extracellular space via excitatory amino acid transporters 1/2 (EAAT1/2) by astrocytes and EAAT2/5 by the presynaptic terminal and then stored into vesicles [17]. In astrocytes, glutamate is converted into glutamine (Gln) by glutamine synthetase and then transported back into the glutamatergic neuron, where it is hydrolyzed into glutamate by glutaminase. Additionally, glycine or d-serine serves as a co-agonist to open the ion channel. If both ligands (glutamate and a co-agonist) bind while the postsynaptic neuron is in a depolarized state, NMDA channels will open, permitting calcium to enter the cells. The increased influx of calcium transform into chemical signals which in turn, leading to the establishment of LTP, a process believed to underlie learning and memory [18]. Overactivation of NMDA receptors resulting in the elevation of intracellular Ca^2+^ levels, which initiates a series of events leading to cell death [19]. In the APPswe-PS1dE9 brain, a mouse model of AD, NMDAR overactived in an age-dependent manner. Memantine, a drug that blocks NMDARs, is widely used in treating AD patients, although its mechanism is still in debate [20].

Regulation of extracellular concentrations of glutamate is also essential to neurons. Over-stimulation can let the synaptic regulation aberrant, leading to alterations in learning and memory, and more serious, neurodegeneration [21]. Glutamate transport systems have the potential to terminate the excitatory signaling, transport glutamate to extrasynaptic receptors, and protect the neurons from excitotoxic injury. The majority of glutamate transport in the CNS, particularly as related to excitatory transmission, is mediated by the sodium-dependent EAATs. When glutamate release surpasses the capacity of astrocyte clearance mechanisms, or expression of EAATs decreased, excitotoxicity can occur. Dysfunction or reduced expression of GLT-1/EAAT-2 has been documented in both chronic and acute neurodegenerative disorders [22].

## Synaptic and extrasynaptic localization of NMDARs

*N*-methyl-d-aspartate receptors subunits differ not only in temporal pattern of expression, but also in cellular localization. NMDARs have been found on both presynaptic and postsynaptic locations on neurons. NMDARs existing on postsynaptic act as detectors for the induction of various forms of short- and long-term synaptic plasticity. NMDARs are also located presynaptically where they mediate synaptic transmission and activity-dependent synaptic plasticity in developing neural circuits [23]. In interneuron–Purkinje cell synapse, the NMDA induce an increase in miniature inhibitory postsynaptic currents (mIPSCs) frequency suggesting the presynaptic NMDARs present on inhibitory axon terminals [24]. In pyramidal cells of the developing visual cortex, presynaptic NMDARs are thought to play a pivotal role in the regulation of spontaneous neurotransmission and induction of timing-dependent long-term depression (tLTD) at pyramidal cell-to-pyramidal cell synapses in layer five. Synapse-specific presynaptic NMDARs activation could provide an important mechanism for governing information processing in neocortical columns during high-frequency firing [25]. In contrast, presynaptic NMDARs expressing on interneuron axon terminals appear to act as coincidence detectors for adjacent glutamatergic and GABAergic activities, leading to coordinated synaptic modification. Moreover, presynaptic NMDARs may play a role in mediating spontaneous transmitter release that can dynamically alter the synaptic efficacy at inhibitory synapses [26].

Extrasynaptic NMDARs are located on dendrites or the sides of spines and also require high glutamate concentrations. In addition, many extrasynaptic NMDARs concentrate at points of contact with adjacent processes including axons, axon terminals, or glia [27]. Many proteins can associate with the NMDARs in these extrasynaptic sites. Extrasynaptic complexes of PSD-95, GKAP and shank, and some of these complexes also contain neuroligin 1. A number of studies suggest that extrasynaptic NMDARs function may depend on the receptor type as well as that of associated proteins [28]. Increasing the extrasynaptic glutamate concentration, including increased stimulation intensities, knockout or inhibition of the glial glutamate transporter can impair LTP and this impairment can be prevented by NMDAR antagonists, suggesting that extrasynaptic NMDARs can obstruct activity-dependent plasticity [29]. Aβ induces glutamate release from astrocytes, which in turn activates extrasynaptic NMDARs on neurons and subsequent decrease in miniature excitatory postsynaptic currents (mEPSCs) which may reflect early synaptic injury [30]. d-serine degradation inhibits synaptic NMDARs, the magnitude of LTP expression is attenuated, while glycine degradation has no effect on LTP, suggesting synaptic NMDARs play a key role in LTP but not extrasynaptic. On the contrary, both synaptic and extrasynaptic NMDARs are necessary for LTD [31].

Activation of synaptic NMDARs mediates synaptic plasticity and is thought to be beneficial for neurons, whereas prolonged activation of NMDARs with subsequent overload of Ca^2+^ entry is deleterious [32]. According to the latest new model, the function of extrasynaptic NMDARs is related to a variety of locations at different distances from the nearest synaptic active zones and arrangements characterized by close association with different kinds of adjacent processes [33]. Synaptic NMDARs are neuroprotective, whereas stimulation of extrasynaptic NMDARs cause loss of mitochondrial membrane potential and cell death. According to this view, it is not the Ca^2+^ overload is the sole determine neurotoxicity, but rather the Ca^2+^ influx through NMDARs located outside the synapse that is particularly harmful to neurons.

## Signaling pathways associated with NMDARs in AD

Many studies have identified key signaling pathways involved in AD pathogenesis. CREB is the prototypical signal-regulated transcription factor, plays an essential role in the maintenance of LTP [34]. Within the hippocampus, CREB-mediated gene expression are associated with synaptic plasticity, learning and memory [35]. Phosphorylation of CREB at residue ser-133 is particularly important for its transcriptional activity, which is decreased in AD [36]. The phosphorylation of CREB can be triggered in neurons by a variety of signaling processes, including an increase in intracellular Ca^2+^ via activation of voltage- or ligand-gated channels such as NMDARs, or activation of receptor tyrosine kinase by growth factors. Calcium entry through synaptic NMDARs induced CREB phosphorylation and BDNF expression, whereas extrasynaptic NMDARs have the opposite effects, trigger the CREB shut-off pathway. Aβ decreases the phosphorylation of CREB through inactivation of PKA and thus inhibits LTP generation [37]. Jacob, a caldendrin binding protein in brain, when localized to the nucleus, attenuates CREB phosphorylation and also promotes a loss of synaptic contacts. Increased NMDAR activity was shown to increase the nuclear accumulation of Jacob [38]. The status of Jacob phosphorylation determines whether it induces cell death or promotes cell survival and enhances synaptic plasticity. Phosphorylated Jacob is translocated to the nucleus after synaptic NMDAR stimulation and is associated with neuroprotection. On the other hand, nonphosphorylated Jacob is translocated after extrasynaptic NMDAR stimulation and is associated with decreased CREB activity, dendritic complexity, and synaptic density [39].

Forkhead transcription factor (FoxO3a) is associated with AD neuropathology and also activated and imported to nuclear by increased extrasynaptic NMDAR activity [40]. Synaptic NMDAR activity suppresses FOXO activity by promoting the Akt-mediated phosphorylation whereas extrasynaptic NMDAR activity stimulates FOXO nuclear import and leads to excitotoxic cell death [41]. Extrasynaptic NMDARs stimulation leads to activation of calpain, cleavage of STEP61 to STEP33, accompanied by increase of p-p38. While synaptic NMDARs activation induces ubiquitination and degradation of STEP61 which correlated with the phosphorylation of ERK1/2 [42]. The ERK1/2 pathway, which can induce activation of CREB has been implicated in NMDAR- mediated neuroprotection. Synaptic NMDARs activity maintains sustained ERK activation, whereas activation of all NMDARs by bath application of NMDA results in ERK activation followed by inactivation. NMDARs exert a bidirectional role in the regulation of ERK based on their localization. Synaptic NMDARs activate ERK whereas the extrasynaptic NMDARs promote ERK inactivation [43]. Thus, synaptic and extrasynaptic NMDARs are mutually antagonistic with regard to ERK signaling.

## Aβ influence NMDAR involved in AD

The cognitive impairment of AD is closely related to synaptic plasticity, in which NMDARs plays a critical role. Activation of NMDARs by Aβ accumulation may occur at early stages of the disease [44]. Aβ, like NMDA evoke an immediately Ca^2+^ rise through activation of GluN2B-containing NMDARs in cultured cortical neurons [19]. Aβ oligomers is reported to impair NMDARs-dependent LTP at hippocampal CA1 and dentate gyrus synapses [45]. And the inhibition of LTP induced by Aβ can be prevented by GluN2B-selective antagonist at low concentrations [46]. Moreover, Aβ-induced LTP impairment in AD can be ameliorated by decreasing extracellular glutamate levels and inhibition of p38MAPK or calpain, which are selectively activated by extrasynaptic NMDARs [47]. NMDARs activation raises Aβ production by increasing a shift from α-secretase to β-secretase activity suggesting that even a mild deregulation of the glutamatergic transmission might lead to Aβ overproduction. In cultured hippocampal neurons, Aβ preconditioning reduces surface expression of NMDAR subunit protein NR1 [48]. Early neuronal dysfunction induced by Aβ is mediated by an activation of NR2B-containing NMDAR in primary neuronal cultures and hippocampal slices from rat and mouse [49]. Moreover, treatment of rat organotypic slices containing pyramidal neurons with Aβ oligomers decrease dendritic spine density and reduce NMDAR-mediated calcium influx into active spines [50]. GluN1 mRNA levels are significantly lower in AD, moreover, GluN2A and GluN2B levels are decreased in susceptible regions of postmortem human AD brain, such as the hippocampus and the cortex [51]. Since GluN2A subunits have been implicated in protective pathways, whereas GluN2B subunits appear to increase neuronal vulnerability. Activation of GluN2A and decrease in GluN2B subunit may be an attempt to reduce Aβ induced neuronal dysfunction [52].

Beta-amyloid reduces glutamatergic transmission and inhibits synaptic plasticity through regulating the number of NMDARs. When applied to cultured cortical neurons, Aβ enhances endocytosis of NMDARs and decreases expression of NMDARs [53]. Consistent with the finding that calcium influx through NMDARs into active spines is reduced by Aβ [50]. Similarly, Aβ decreases synaptic glutamatergic currents in rat hippocampal neurons. Thus, Aβ appears to regulate the synaptic NMDARs, disrupting the balance between synaptic and extrasynaptic NMDAR signaling. The expression of glutamate transporters is reduced significantly both in human tissue from AD patients [54] and animal models of AD [55].

## Tau implicate in excitotoxicity

Like Aβ, tau has also been implicated in glutamate excitotoxicity and synaptic dysfunction. Tau phosphorylation results in mislocalization of tau from axons to dendritic spines and decreased expression of AMPA receptors and LTP deficits, and subsequently synaptic impairment [56]. The acute overexpression of tau phosphorylation increases NMDAR transmission and thereby facilitation of LTD. Phosphorylation of tau can be induced by NMDA receptor stimulation as well as by incubation with Aβ. However, the acute NMDA receptor-dependent tau phosphorylation is reversible whereas tau phosphorylation after 5 days of Aβ exposure is not [57]. Fyn is a tyrosine kinase that phosphorylates the NR2B receptor subunit, thereby stabilizing its interaction with PSD95 in dendritic spines. Tau mediates the binding and transport of Fyn to dendritic spines. Tau reductions cannot transport Fyn to dendritic spines, prevent the memory deficits and excitability caused by Aβ [58]. Endogenous tau is found at postsynaptic sites where it binds not only Fyn but also the PSD95-NMDAR complex. NMDARs activation induces phosphorylation tau at specific sites, which regulate the interaction of tau with Fyn and the PSD95-NMDAR complex. Tau phosphorylation also increases NMDA receptor transmission and facilitation of LTD [57]. Overexpression of human tau in primary neuronal cultures causes cell death, which is inhibited by ifenprodil, a GluN2B selective antagonist, suggesting that tau-induced neurotoxicity by stimulation of extrasynaptic NMDARs containing GluN2B subunit. Calpain, activated by Ca^2+^ influx through NMDAR, degrades tau protein into a toxic N-terminal peptides which leading to cell death [59]. Memantine treatment preferentially blocks E-NMDARs, attenuates tau phosphorylation and excitotoxicity [60]. GluN2A-containing NMDA receptors modulate tau phosphorylation in rat hippocampal slices. GluN2A-contained NMDA receptor induces reduction of tau phosphorylation by a PKC/GSK3 pathway [61]. Although the investigation into the role of tau in glutamate excitotoxicity is a relatively nascent field, tau play a role in mediating Aβ-induced neuronal death and can induce synaptic dysfunction independently [62]. Thus, further elucidation of tau-dependent and tau-independent pathways is needed, as well as a better understanding of NMDARs in various pathological effects.

## Treatment strategies for AD targeting NMDARs

Several studies reviewed above demonstrate the importance of NMDARs in AD progression indicating that targeting NMDARs seem to be a promising therapeutic treatment for AD. Memantine is a NMDA receptor antagonist with low-affinity that has been approved by the FDA for treatment of AD patients. Memantine was shown to prevent neuronal necrosis, disruption of axonal transport trafficking, DNA fragmentation and neurite retraction. Memantine unable to attenuate Aβ-induced potentiation of extracellular glutamate levels but protects neurons by attenuating tauphosphorylation [60]. Moreover, in 3xTg-AD mice, memantine treatment ameliorates cognitive impairment and reduces Aβ accumulation and tau phosphorylation [63]. In clinical trials, memantine has shown significant benefits in aspects of cognition such as language, memory, praxis, functional communication and in activities of daily living for AD patients [64]. Neramexane, a non-competitive NMDAR antagonist, has shown to be efficient in enhancing long-term memory in adult rats as memantine, suggesting that this antagonist may have a potential therapeutic applications [65]. Ifenprodil, a specific NR2B receptor antagonist, prevents Aβ-induced endoplasmic reticulum stress and hippocampal dysfunction and microtubule deregulation as well as Ca^2+^ rise [66]. Ifenprodil also prevents Aβ-induced inhibition of LTP, impairment of synaptic transmission and retraction of synaptic contacts [49]. In acute hippocampal slices, the selective NR2B antagonists ifenprodil or Ro25-6981 were also efficient to rescue of LTP inhibition by soluble Aβ. These results suggest that targeting GluN2B subunit of NMDARs may be another way to prevent AD progression.

Repetitive transcranial magnetic stimulation (rTMS) is a non-invasive method of brain stimulation which has been investigated in several neuropsychiatric disorders, such as AD, Parkinson’s disease and schizophrenia and as a potent therapeutic tool in depression. rTMS enhance the LTP at hippocampal CA3-CA1 synapses and increase NR1 and NR2B expression resulting in improving spatial learning and memory abilities in the rat model of vascular dementia [67]. Low-frequency rTMS reverse the decreased NMDAR levels and deficits in LTP and spatial memory in Aβ induced AD mouse models [68]. rTMS treatment also improve the cognitive ability and language performance in AD patients suggesting that rTMS may be a effective therapeutic technique in the treatment of neurodegenerative diseases [69].

## Conclusions

Glutamatergic dysfunction linked to Ca^2+^ dyshomeostasis plays a great importance in pathophysiology of AD. Changes in NMDARs appear to be involved in synaptic dysfunction in early stages of AD. Recent researches reveal that in particular the tripartite glutamatergic synapses play an important role in the pathophysiology of AD, which provide us a better understanding of this complex and critical cross-talk mediated by glutamate. These insights help us to develop new and potentially more effective compounds as therapeutics for AD. Moreover, differential regulation of synaptic and extrasynaptic NMDARs seems to underlie distinct neuronal fates, either inducing cell survival or cell death. In this regard, selective inhibition of NMDARs-mediated excitotoxicity alone may help to slow down the progression of synaptic disruption in AD. Unfortunately, these therapeutics do not trigger a complete cure or an improvement in life expectancy when applied in late stage AD. Thus implementation of earlier therapeutic strategies targeting NMDARs and the intricate signaling pathways is needed.

## Abbreviations

NMDARs: *N*-methyl-d-aspartate receptors
AD: Alzheimer’s disease
Aβ: Beta-amyloid
NFTs: Neurofibrillary tangles
CNS: Central nervous system
VGLUTs: Vesicular glutamate transporters
EAATs: Excitatory amino acid transporters
LTP: Long-term potentiation
CREB: cAMP response element binding protein
